# THE MODERATING RELATIONSHIP BETWEEN FUTURE SELF-CONTINUITY AND NUMERACY ON RETIREMENT SAVINGS

**DOI:** 10.1093/geroni/igae098.3747

**Published:** 2024-12-31

**Authors:** Laken Mooney, Ryan Best

**Affiliations:** West Virginia University, Morgantown, West Virginia, United States; West Virginia University, Morgantown, West Virginia, United States

## Abstract

Worldwide, lifespan is increasing while birth rates are declining resulting in an increasing proportion of older adults in the population. This demographic shift will manifest in an increased proportion of adults in retirement who will rely on non-employment sources of income. Though Social Security provides a key source of retirement income, it is often insufficient and requires supplementation by other sources. Since the late 1970s, the most common retirement savings programs have largely shifted from employers (e.g., pensions) to individuals (e.g., 401k and IRA programs). As such, a greater responsibility for retirement savings now falls on individuals and is more susceptible to variance in individual decision making. Individual difference factors which predict retirement savings include numeracy, the ability to understand and work with numbers, and future self-continuity (FSC), the sense of connectedness we have to our future selves. Using a zero-inflated Poisson model to analyze data from the RAND American Life Panel (n=798), we found a significant interaction between numeracy and FSC when predicting retirement savings (p<.001) after controlling for age and income. Among those that had retirement savings, increased numeracy predicted greater savings for individuals with high, but not low, FSC. This demonstrates that highly numerate individuals appear to have the skills necessary to maximize retirement savings but may not utilize these skills unless they have a strong connection to their future self. Results are discussed in light of the current landscape of retirement preparation and how to best promote individual saving.

